# Class II Transactivator (CIITA) Enhances Cytoplasmic Processing of HIV-1 Pr55Gag

**DOI:** 10.1371/journal.pone.0011304

**Published:** 2010-06-24

**Authors:** Kristen A. Porter, Lauren N. Kelley, Annette George, Jonathan A. Harton, Karen M. Duus

**Affiliations:** Center for Immunology and Microbial Disease, Albany Medical College, Albany, New York, United States of America; Karolinska Institutet, Sweden

## Abstract

**Background:**

The Pr55*^gag^* (Gag) polyprotein of HIV serves as a scaffold for virion assembly and is thus essential for progeny virion budding and maturation. Gag localizes to the plasma membrane (PM) and membranes of late endosomes, allowing for release of infectious virus directly from the cell membrane and/or upon exocytosis. The host factors involved in Gag trafficking to these sites are largely unknown. Upon activation, CD4+ T cells, the primary target of HIV infection, express the class II transcriptional activator (CIITA) and therefore the MHC class II isotype, HLA-DR. Similar to Gag, HLA-DR localizes to the PM and at the membranes of endosomes and specialized vesicular MHC class II compartments (MIICs). In HIV producer cells, transient HLA-DR expression induces intracellular Gag accumulation and impairs virus release.

**Methodology/Principal Findings:**

Here we demonstrate that both stable and transient expression of CIITA in HIV producer cells does not induce HLA-DR-associated intracellular retention of Gag, but does increase the infectivity of virions. However, neither of these phenomena is due to recapitulation of the class II antigen presentation pathway or CIITA-mediated transcriptional activation of virus genes. Interestingly, we demonstrate that CIITA, apart from its transcriptional effects, acts cytoplasmically to enhance Pr160*^gag-pol^* (Gag-Pol) levels and thereby the viral protease and Gag processing, accounting for the increased infectivity of virions from CIITA-expressing cells.

**Conclusions/Significance:**

This study demonstrates that CIITA enhances HIV Gag processing, and provides the first evidence of a novel, post-transcriptional, cytoplasmic function for a well-known transactivator.

## Introduction

HIV polyprotein Gag serves as a scaffold to promote assembly of progeny virions at cellular membranes [1] and recruits components of the vesicular protein sorting pathway to facilitate virus budding [2], [3], [4]. Concomitantly, the virally encoded protease begins to cleave Gag, which is required for complete virion maturation and infectivity [5], [6], [7]. Gag proteins can be detected at both the PM and the membranes of endosomes among different cell types, suggesting that budding is not limited to one cell-type specific locale [8], [9], . Further, host factors which participate in targeting Gag trafficking to particular membranes are largely unknown. As Gag and infectious virus can originate from two cellular locations, two models for Gag trafficking have emerged. The first model proposes that following synthesis, Gag traffics to endosomal membranes, and upon exocytosis is deposited on the PM, where it serves as the site for productive virus assembly [14], [17]. The second model proposes that Gag is first trafficked to the PM, where virus assembly occurs, and then excess Gag is internalized to intracellular compartments [14], [18], [19], [20], that serve as sites of productive virus assembly [15], [21].

MHC class II heterodimers follow a similar trafficking route, appearing at both the PM and specialized multivesicular bodies (MVBs) called MHC class II containing compartments (MIICs) [22]. MHC class II is utilized by antigen presenting cells (APCs) to present exogenous processed antigen to CD4+ T cells [22], [23], [24]. MHC Class II genes, including: HLA-DR, -DP and –DQ and the accessory molecules, invariant chain (Ii) and HLA-DM, are transcriptionally activated by the class II transactivator (CIITA), the global regulator of coordinate class II MHC gene expression [25], [26]. As CIITA is induced in CD4+T cells upon activation, these cells express MHC class II [27], [28]. Upon synthesis, HLA-DR heterodimers are assembled in the ER and the immature complex (HLA-DR+ Ii) travels through the secretory pathway to MIICs, where the specialized HLA-DM chaperone loads the HLA-DR heterodimer with peptide [22], [29], [30]. Interestingly, both immature and mature forms of HLA-DR can be found at the PM and can be subsequently internalized to MIICs due to a di-leucine motif in the cytoplasmic tail of Ii (immature HLA-DR) and a di-leucine motif and/or ubiquitination of conserved lysine residues within the HLA-DR β chain (mature HLA-DR), respectively [22], [29], [31], [32], [33], [34], [35], [36]. Therefore, a connection between HLA-DR and Gag trafficking would not be surprising as both have an alternative route to intracellular compartments by way of the PM. Indeed, expression of HIV-1 Nef, Vpu and Gag have been shown to alter HLA-DR trafficking [37], [38], [39], [40]. In addition, HLA-DR is preferentially acquired on the viral envelope of budding virions, which enhances virion infectivity and may play a role in bystander apoptosis of T lymphocytes [41], [42], [43]. Therefore, HLA-DR localization at virus assembly sites is not unexpected.

Finzi *et al*. (2006) addressed the contribution of MHC class II-induced MIIC formation to Pr55Gag trafficking by monitoring virus budding in the presence of transiently expressed HLA-DR heterodimers [8]. In HEK-293T cells expressing HLA-DR, there was a marked redistribution of Gag to intracellular compartments, and reduced virus release at the PM; mature virus budded into HLA-DR containing multivesicular bodies and was retained [8]. We hypothesized that recapitulating endogenous expression of the entire class II antigen presentation pathway in producer cells via expression of CIITA would restore infectious virus release and provide a more physiologically relevant model for HIV-1 assembly studies. As expected, stable or transient expression of CIITA did not induce intracellular retention of Gag. However, HLA-DR induced Gag retention could not be alleviated by transient co-expression with HLA-DM and/or Ii, or CIITA-mediated upregulation of the recycling endosome GTPase, Rab4B [44]. Further, mutating ubiquitinatible lysine residues or complete truncation of the cytoplasmic tails of the HLA-DR α and β chains did not restore virus release. Rather, limiting the amount of HLA-DR DNA transfected into cells restored virus release and alleviated Gag retention. Curiously, CIITA expressing cells produced virus that was significantly more infectious than CIITA deficient cells, and this was independent of the class II antigen presentation pathway. CIITA enhances infectivity of virions from producer cells through a novel function, by improving maturation through an increase in viral protease-dependent Gag processing. Using a panel of CIITA mutants, we demonstrate that cytoplasmic CIITA increases Gag-Pol polyprotein levels. Overall, our work reveals a novel cytoplasmic, post-transcriptional function of CIITA, which is expressed upon T cell activation and is constitutively expressed in macrophages and dendritic cells, all known targets of HIV infection.

## Results

### CIITA expression does not induced HLA-DR-mediated Gag retention

**Figure 1:**
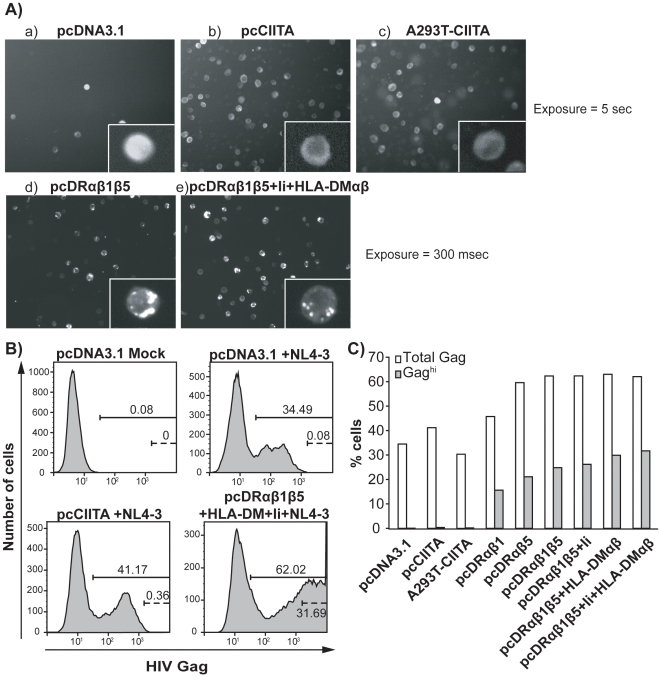
CIITA does not induce HLA-DR-mediated Gag retention. A293T cells were co-transfected with indicated plasmids and HIV-1_NL4-3_ and following intracellular staining of Gag (using an antibody which recognizes the CA p24 domain of Gag), immunofluorescence microscopy (A) and flow cytometry (B) were performed. Total levels of Gag present in cells as well as cells which contain dense staining of Gag (Gaghi) are depicted (C). Data are representative of three independent experiments.

Transient expression of the class II heterodimer, HLA-DR, induces intracellular accumulation of Gag and a marked reduction in extracellular virus release from producer cells [8]. This previous study focused on HLA-DR in the absence of Ii and HLA-DM, critical components of the antigen processing and presentation pathway which influence MHC class II trafficking. To determine if expression of the complete MHC class II pathway could overcome Gag and virus retention we used CIITA to coordinately express these genes. Cells were either transiently (pcCIITA) or stably (A293T-CIITA) transfected with CIITA (Figure S1) and HIV-1_NL4-3_ DNA, and Gag localization was monitored by immunofluorescence and compared to cells transiently expressing the HLA-DR α and β heterodimers (pcDRαβ1β5), or vector-only (pcDNA3.1). As expected, a weak but uniform Gag signal was present in vector-only transfected cells (Figure 1Aa) and transient HLA-DR expression induced a marked redistribution of Gag as indicated by a dense, punctuate Gag staining pattern (Figure 1Ad). Conversely, Gag accumulation was not observed when CIITA was either transiently or stably expressed (Figure 1Ab or c, respectively). Therefore, transient expression of HLA-DR in producer cells does indeed lead to Gag retention, which is not observed in the presence of CIITA. However, recapitulation of the MHC class II pathway in *trans* (pcDRαβ1β5+Ii+HLA-DMαβ), also resulted in dense accumulation of Gag signal (Figure 1Ae), suggesting that CIITA-mediated coordinate activation of HLA-DR, -DM and Ii expression is insufficient to overcome Gag retention. Flow cytometric analysis confirmed these findings, as cells transfected with HLA-DR heterodimers and/or co-transfected with some or all of the components of the class II antigen presentation pathway stained as Gag^hi^, indicating Gag accumulation (Figure 1B and C). However, this population was absent in cells transiently or stably expressing CIITA (Figure 1B and C). Therefore, absence of Gag accumulation in CIITA expressing cells is likely not due to its transactivation of the MHC class II antigen presentation pathway.

### CIITA increases virion infectivity independently of HLA-DR and the class II pathway

**Figure 2:**
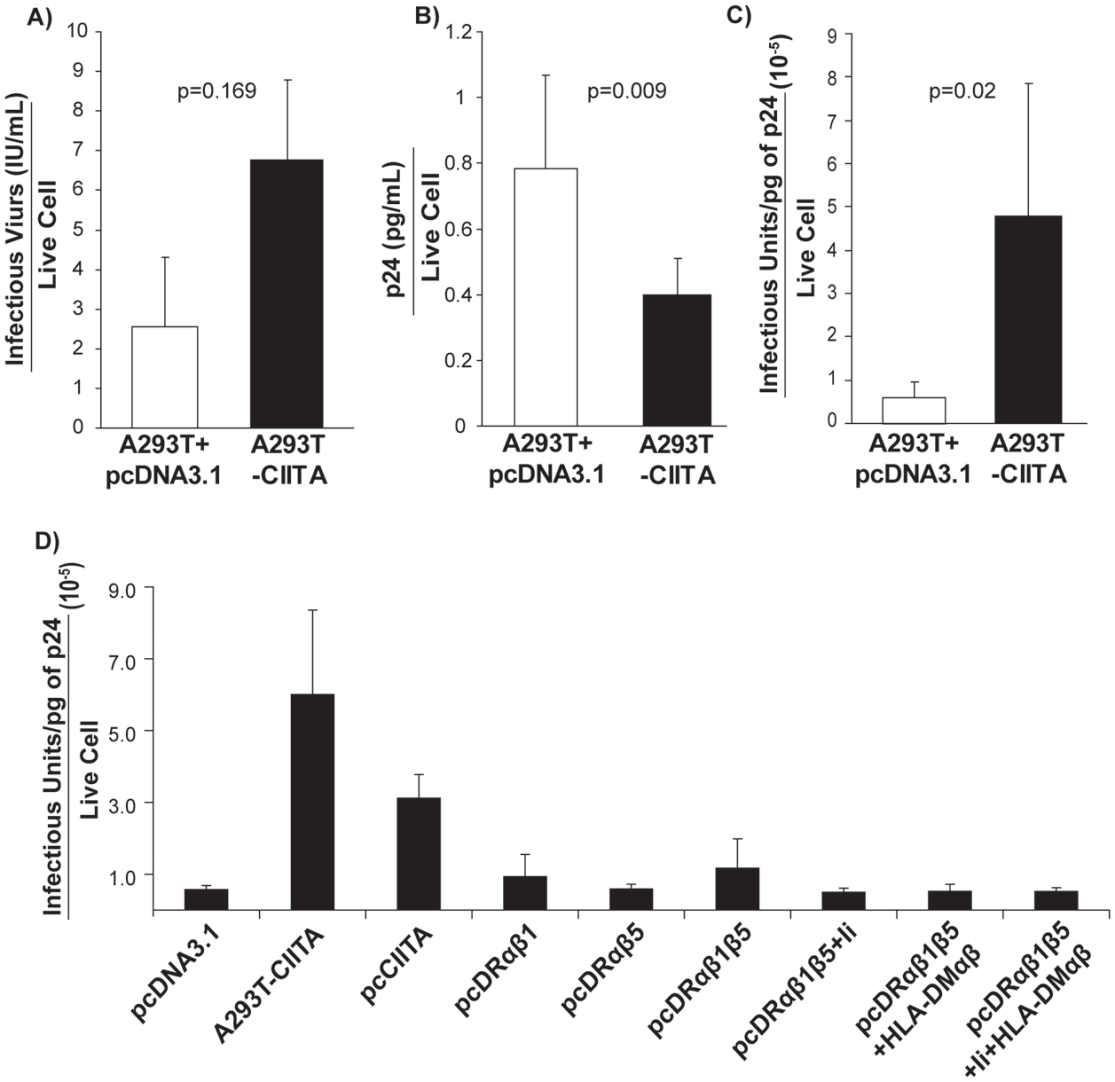
CIITA increases virion infectivity independently of HLA-DR and the class II pathway. Producer cells were transfected with HIV-1_NL4-3_ and the indicated plasmids (A293T-CIITA is a stable clone) and at 48 h.p.t. infectious virus (A) and virus particle release (B) was determined by a GHOST infectivity assay and p24 ELISA, respectively. The number of infectious virions per total number virions released from a live cell was also determined (C). Similarly, the infectivity of virions from cells expressing HIV-1_NL4-3_ and the indicated constructs was determined (D). Standard deviation of the mean for ≥5 independent experiments is presented. Statistical significance (p≤0.05) was determined by a two-sample unequal variance student's T-test.

To assess whether CIITA-mediated alleviation of Gag retention in class II expressing cells correlated with restored virus production, virus particle and infectious virus release were measured by p24 ELISA and GHOST cell infectivity assays, respectively. Virus release, both infectious and particle titers) were reduced when cells were transfected with either HLA-DR or other components of the MHC class II antigen presentation pathway (Figure S2), confirming a correlation between Gag retention and reduced virus titers in the presence of HLA-DR, as previously demonstrated [8]. These assays also demonstrate a dramatic increase in infectious virus release from CIITA-expressing cells as compared to cells expressing vector only (Figure 2A & S2), despite significantly fewer virus particles being released from a CIITA-expressing cell (as measured by pg/mL of p24 in the culture supernatant, Figure 2B & S2). Therefore, while fewer virus particles are released from a CIITA- expressing cell, those particles are significantly more infectious (Figure 2C).

Previously, HLA-DR incorporation into the envelope of budding virions was demonstrated to enhance virion infectivity [45], [46]. To determine if CIITA-enhancement of virion infectivity was due to HLA-DR or other components of the class II antigen presentation pathway, we measured virion infectivity from cells expressing these proteins. However, virions from these cells were just as infectious as virions released from vector only controls, yet not as infectious as virions from cells either stably or transiently-expressing CIITA (Figure 2D). Therefore, CIITA enhancement of virion infectivity is independent of the MHC class II antigen presentation pathway. Together, these data suggest CIITA has two effects on the HIV replicative cycle in producer cells, both of which are independent of the MHC II antigen processing pathway; i) it does not induce HLA-DR, mediated intracellular retention of Gag and ii) it increases the infectivity of HIV virions.

### Rab4B does not alleviate HLA-DR mediated Gag retention

**Figure 3:**
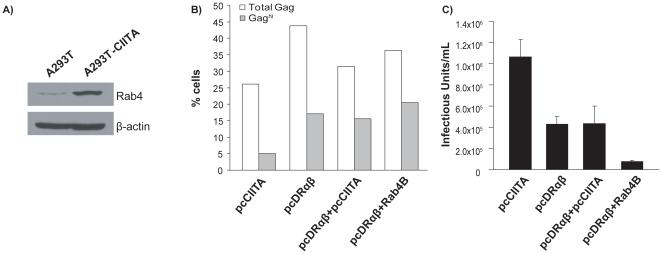
Rab4B does not alleviate HLA-DR mediated Gag retention. The level of Rab4 expressed in CIITA-expressing A293T cells was determined by immunoblotting, where β-actin served as a loading control (A). Cells were co-transfected with the indicated constructs and HIV-1_NL4-3_ and Gag retention was monitored by intracellular staining and flow cytometry (B) and infectious virus release was measured via GHOST infectivity assays (C). Data are representative of three independent experiments and error bars indicate standard error.

CIITA has been shown to upregulate expression of Rab4B, a small GTPase involved in endocytic recycling [44], [47]. Therefore, we hypothesized that CIITA via the action of Rab4B, could increase Gag recycling back to the PM, thereby alleviating HLA-DR-mediated Gag retention. Commercially available Rab4 antibodies cannot distinguish between Rab4A and 4B. However, Krawczyk *et al*. (2007) demonstrated by chromatin immunoprecipitation that CIITA specifically associates with the Rab4B and not the Rab4A promoter [44]. Immunoblotting demonstrated that there is an increase in Rab4 in CIITA-expressing cells (Figure 3A), therefore it is likely that this is in the form of Rab4B. However, when Rab4B and HLA-DR were co-expressed in producer cells with the HIV-1_NL4-3_ plasmid, it did not rescue intracellular retention of Gag (Figure 3B). In fact, infectious virus release from cells transiently co-expressing Rab4B and HLA-DR was further reduced, suggesting that Rab4B does not alleviate HLA-DR-mediated retention of Gag (Figure 3C).

Up until this point, we had not co-expressed CIITA with HLA-DR in producer cells to determine if CIITA could alleviate retention of Gag and/or restore infectious virus release as would be expected. Interestingly, when CIITA was transiently expressed with the HLA-DR heterodimers in producer cells, Gag still accumulated intracellularly and infectious virus production remained reduced (Figure 3B and C, respectively), suggesting that expression of CIITA is not sufficient to compensate for HLA-DR-mediated Gag retention. However, when HLA-DR is endogenously expressed under the control of CIITA, retention is alleviated.

### Gag retention is an artifactual effect of HLA-DR overexpression

**Figure 4:**
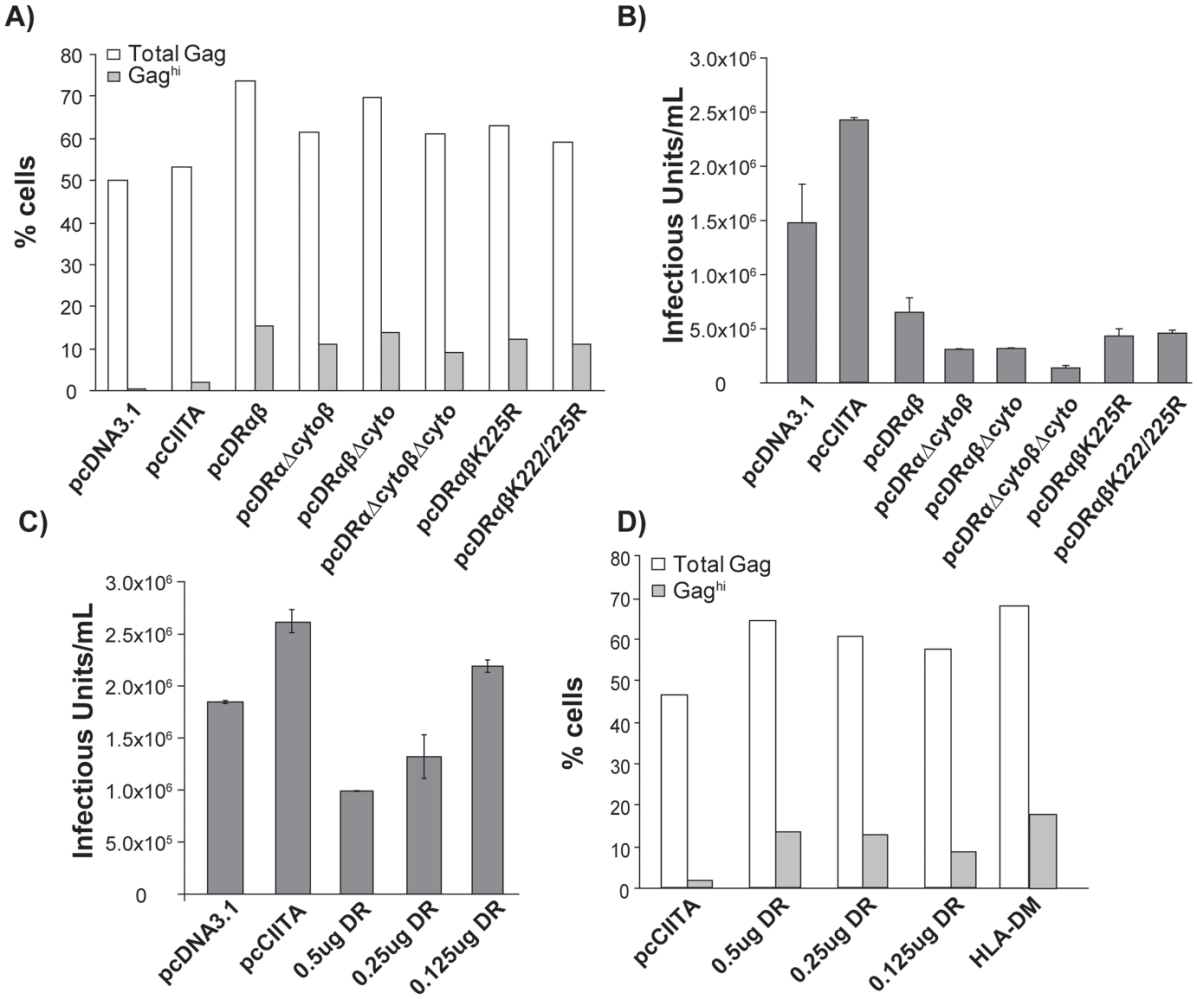
MHC class II- induced Gag retention is a consequence of HLA-DR overexpression in producer cells. Mutations were made in the HLA-DR cytoplasmic tails as indicated and transfected into producer cells at a 1∶1 ratio with HIV-1_NL4-3_ and Gag retention by flow cytometry (A) and infectious virus release (B) was monitored. Decreasing amounts of HLA-DR α and β heterodimer constructs were transfected into producer cells with HIV-1_NL4-3_ and infectious virus release (C) and Gag retention (D) were monitored. Data are representative of three independent experiments and error bars indicate standard error.

Previously, Finzi *et al*. (2006) demonstrated that Gag retention in HLA-DR+, multivesicular bodies could be alleviated when the cytoplasmic tails of the HLA-DR α and β chains were removed [8]. Ubiquitination of a conserved lysine residue on the HLA-DR β chain induces intracellular accumulation of class II heterodimers in MHC class II compartments (MIICs) [35], [36]. Thus, the ubiquitination state of HLA-DR, might influence Gag retention. To address this idea, Lys225Arg (pcDRαβK225R) and Lys222/225Arg (pcDRαβK222/225R) point mutations were made in the HLA-DR beta chain. Despite these mutations, HLA-DR expression on the surface of A293T cells was not significantly altered (flow cytometric data not shown), nor was intracellular retention of Gag alleviated or infectious virus release restored (Figure 4A and B, respectively). Therefore, we attempted to alleviate Gag retention and restore infectious virus release, as demonstrated by Finzi *et al*. (2006) [8], by complete truncation of the cytoplasmic tails of both HLA-DR α and β (pcDRαΔcyto and pcDRβΔcyto, respectively). However, loss of either cytoplasmic tail (pcDRαΔcytoβ and pcDRαβΔcyto), or both cytoplasmic tails (pcDRαΔcytoβΔcyto), from the heterodimer did not alleviate Gag retention and even further reduced infectious virus release from these cells (Figure 4A and B, respectively). Our results may differ from Finzi's because of the differences in cell type, or HLA-DR gene alleles, nevertheless they strongly suggest that transient HLA-DR expression in producer cells induces Gag retention. Further, flow cytometry to monitor surface HLA-DR expression demonstrates that transient transfection of HLA-DR induces an approximate half-log to log-fold increase in HLA-DR as compared to cells stably or transiently expressing CIITA, respectively (Figure S1). Therefore, it is possible that HLA-DR induced Gag retention is a consequence of HLA-DR overexpression in this transient system and thus endogenous expression of HLA-DR via CIITA does not result in the same phenotype.

To test this possibility, virus producer cells were transfected with increasing amounts of HLA-DR in the presence of HIV-1_NL4-3_ DNA, and infectious virus release was monitored. As HLA-DR expression increased, the level of infectious virus release decreased (Figure 4C). Further, while not statistically significant, there was a positive correlation (r = 0.9954) between Gag accumulation (Gag^hi^) and increasing HLA-DR expression (Figure 4D), suggesting Gag retention is likely related to HLA-DR overexpression. Similarly, when HLA-DM, which is structurally similar to HLA-DR, was overexpressed in producer cells, Gag retention was also induced (Figure 4D) and infectious virus release reduced (data not shown). As endogenous expression of the class II antigen presentation pathway by CIITA does not induce Gag retention and limiting expression of HLA-DR or –DM likewise has a limited effect on Gag retention in producer cells, these data collectively suggest that overexpression of the class II pathway alters Gag trafficking in producer cells leading to reduced infectious virus release. However, these data do not provide an explanation for the increased infectivity of virions released from CIITA-expressing cells.

### CIITA expression enhances virion maturation through increased Gag processing

**Figure 5:**
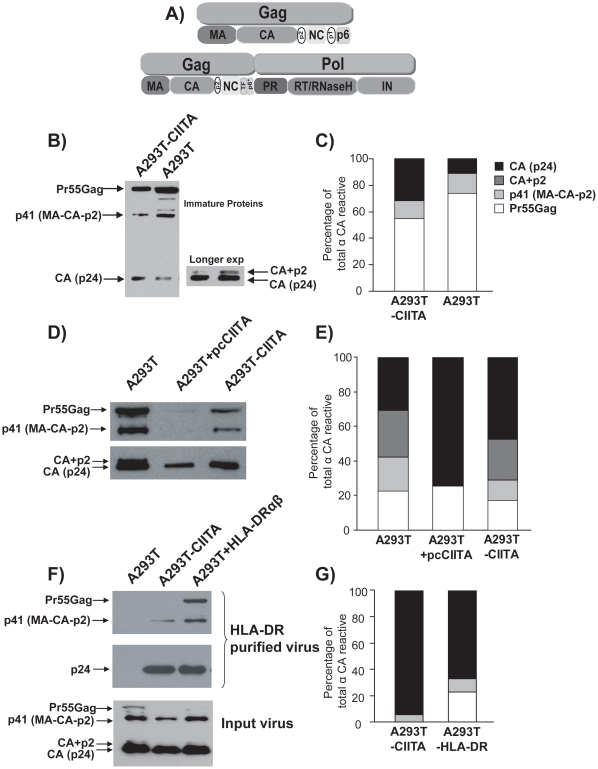
CIITA enhances virion maturation through increased Gag processing. Diagram of Pr55Gag and Pr160Gag-Pol and their respective cleavage products (A). Protein lysates from cell equivalents of HIV-1_NL4-3_ transfected vector-only (A293T) or CIITA expressing (A293T-CIITA) cells (B) were western blotted with an antibody which recognizes CAp24 of Gag. Similarly, cell-free virions from vector-only (A293T), transient-CIITA expressing (pcCIITA) or stably-expressing CIITA (A293T-CIITA) (D), or virions affinity purified with biotinylated-anti-HLA-DR antibody (L243) on streptavidin-coated magnetic beads (F, top panel, where bottom panel is input control virus), were western blotted for Gag processing. Densitometric analysis of western blots was performed and quantitative analysis of Gag cleavage products is presented as percentage of total reactive bands (C, E and G). Data are representative of three independent experiments.

Viral protease cleavage of the Gag and Gag-Pol polyproteins is required for virion maturation and infectivity [5], [6]. The mature complement of HIV-1 structural proteins includes matrix (MA [p17]), capsid (CA [p24]), nucleocapsid (NC[p7]) and p6, as well as spacer peptides, SP1 [p2] and SP2 [p1] within Gag (Figure 5A). The enzymatic proteins, integrase (IN[p32]), reverse transcriptase (RT [p66/51]) and protease [p10], as well as the transframe octapeptide (TFP) and p6* are generated from the Gag-Pol polyprotein (Figure 5A) [13], [48], . As there is an increase in virion infectivity from CIITA-expressing cells, we hypothesized that the processing of Gag polyproteins may be enhanced in these cells. As suspected, analysis of cell lysates from equal numbers of HIV-1 transfected A293T-CIITA and A293T cells demonstrated a higher ratio of mature CAp24 to Pr55Gag in CIITA-expressing cells (Figure 5 B&C), demonstrating that Gag processing in the presence of CIITA is enhanced. Additionally, increased levels of processing intermediates are present in A293T cell lysates. Specifically, the p41 (MA-CA-SP1) and p25 (CA+p2) products were increased (Figure 5 B&C) in these cells, indicating that Gag processing in the presence of CIITA is more efficient. Gag processing is also enhanced in cell-free virions from cells either transiently or stably expressing CIITA, as demonstrated by the increased levels of CAp24, and the loss of the p41 and p25 intermediate products relative to Pr55Gag (Figure 5D&E). Collectively, these results suggest that CIITA increases Gag polyprotein processing, leading to enhanced production of infectious virions.

### HLA-DR does not increase Gag processing

HLA-DR is incorporated into the HIV virion envelope [52] and is transcriptionally activated by CIITA, therefore its contribution to Gag processing was assessed. Virions from HLA-DR-expressing cells exhibited less Gag processing than virions from CIITA-expressing cells (Figure 5F, bottom panel). When virions from both cell lines were affinity-purified on HLA-DR binding columns, Gag processing was reduced in those produced in the HLA-DR-expressing cells as compared to virions from cells which express CIITA (Figure 5F, upper panel and 5G), suggesting that HLA-DR alone does not contribute to increased Gag processing. Similar to our previous results, there are fewer processing intermediates present in virions from CIITA-expressing cells, suggesting that CIITA expression enhances virion infectivity by increasing maturation through more complete Gag processing.

### CIITA enhancement of Gag processing is through the viral protease activity

**Figure 6:**
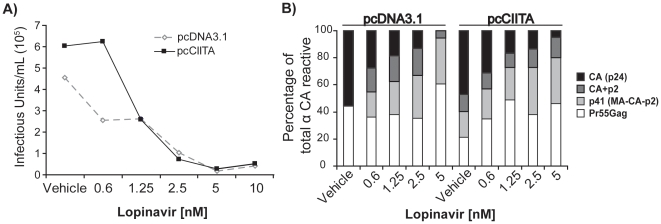
CIITA enhancement of Gag processing and viral infectivity is through the viral protease. Following co-transfection with vector-only (pcDNA3.1) or pcCIITA and HIV-1_NL4-3_, producer cells and were treated with indicated concentrations of Lopinavir and 17 h later infectious virus titers were determined by GHOST assay (A) and Gag processing was measured via western blotting with antibody against CAp24 followed by densitometric analysis of Gag cleavage products (B). Data are representative of three independent experiments.

Cleavage of the Gag and Gag-Pol polyprotein is mediated specifically by the virally-encoded protease [53], [54], [55]. We considered that CIITA might increase viral protease processing of Gag; thus, we examined Gag processing and infectious virus release from CIITA-expressing HIV producer cells in the presence of a protease inhibitor. If CIITA is increasing protease processing, such an enhancement should be overcome by lower doses of the HIV protease inhibitor, Lopinavir. As expected, between concentrations of 0.6 and 1.25 nM of Lopinavir infectious virus release from CIITA-expressing cells was reduced to that of A293T cells treated with vehicle only (Figure 6A). To determine if the reduced Gag processing correlated with decreased infectious virus release, we monitored Gag cleavage by western blotting and as expected, between 0.6 and 1.25 nM of Liponavir, Gag processing in virions from CIITA-expressing cells was returned to that of vehicle treated, vector-only control cells (Figure S3). These results directly demonstrate that CIITA-mediated enhancement of Gag processing and infectious virus release is through the HIV protease.

### CIITA acts cytoplasmically to improve and enhance Gag processing

**Figure 7:**
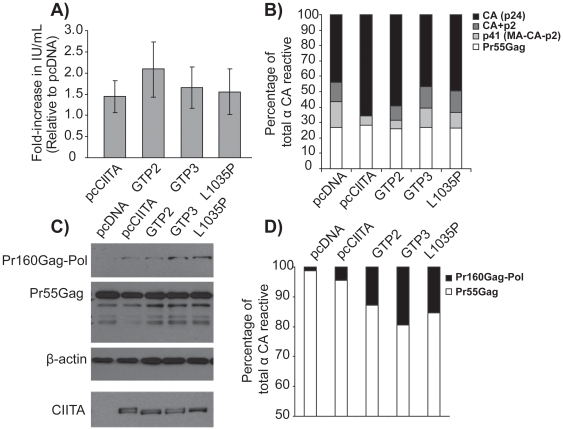
CIITA cytoplasmically increases Pr160Gag-Pol levels. Producer cells were transfected with the indicated CIITA constructs and HIV-1_NL4-3_; 48 h later infectious virus titers were determined by GHOST assay (A, n>2, error bars rep. standard deviation of the mean). Gag processing was measured via western blotting with antibody against CAp24 and densitometric analysis of Gag cleavage products (B). Producer cells were transfected with indicated constructs and then 16 hours later with HIV-1_NL4-3_ before treatment with 80 nM Lopinavir at 4.5 hours post-transfection. 17 hours later, cell lysates were blotted with the indicated antibodies (C) and western blotting with antibody against CAp24 followed by densitometric analysis allowed for the determination of the percentage of Gag-Pol and Gag, following normalization to β-actin (D). Unless indicated, data are representative of three independent experiments.

The only known function of CIITA is as a transcriptional co-activator; therefore, we thought it likely that it drives the expression of a gene which enhances viral protease-mediated Gag processing. Therefore, we reasoned that expression of CIITA mutants which fail to localize to the nucleus should not mediate increased Gag processing or enhanced infectious virus release. To test this idea, a panel of CIITA mutants (GTP2, a magnesium ion coordination site mutant and GTP3, a guanine ring-binding domain mutant and L1035P, a point mutant in the C-terminal leucine rich repeat domain, which are all defective for nuclear localization and fail to activate HLA-DRA transcription [56], [57], [58]), were individually co-expressed with HIV-1_NL4-3_ DNA, and infectious virus release and Gag processing were monitored. Interestingly, producer cells expressing these mutants also produced more infectious virus as compared to vector-only control (Figure 7A) and had increased Gag processing in both cell-bound virions and cell-free virions (Figure 7B and Figure S4, respectively). Loss of CIITA nuclear localization (and therefore coactivation potential), did not hinder increased Gag processing and infectious virus release, strongly suggesting that CIITA acts cytoplasmically to increase HIV virus maturation independent of its transactivation function. We further evaluated CIITA transactivation potential as a mechanism of enhanced virus release utilizing a different model system. NIH 3T3 BALB/c cells (expressing human p32 to allow for virus like particle production [59]) expressing CIITA and transfected with an LTR-deficient HIV genome construct (pcHIV PAL),under control of a CMV promoter had a dramatic increase in virus-like particle production as compared to NIH 3T3 cells in the absence of CIITA (Figure S5). This result provides further evidence that CIITA enhancement of virus production is independent of its transactivation potential on the HIV LTR.

### CIITA increases the viral protease through increased Gag-Pol levels

To determine the mechanism by which CIITA mediates protease activity in these cells, we analyzed the expression of the Gag and Gag-Pol in CIITA-expressing cells. Expression of the HIV protease is extremely toxic to cells, and is thus very tightly regulated during virus replication [60], [61], [62]. The viral protease arises from the alternative Gag translation product (Figure 5A), Gag-Pol which, accounts for only 2–10% of Gag polyproteins and results from ribosomal frameshifting at a conserved heptamer “slippery sequence” and a secondary stem-loop structure on *gag-pol* transcripts [51], [63]. Therefore, increased viral protease-mediated processing of the Gag polyproteins from CIITA-expressing cells may be due to an increase in overall viral protease levels, due to increased Gag-Pol synthesis. To establish the ability of CIITA to mediate the enhanced production of HIV protease, cells were transiently co-transfected with either CIITA or pcDNA and HIV-1_NL4-3_ DNA, and were treated with 80 nM of Lopinavir to inhibit the majority of Gag polyprotein processing. Gag-Pol levels relative to Gag were then determined by western blotting. While 1–2% of all Gag polyprotein in cell lysates of vector-only cells was in the form of Gag-Pol, expression of CIITA increased this level to almost 5% (Figure 7C and D), indicating that CIITA increases Gag-Pol levels in virus producer cells. We also measured the potential of the cytoplasmic CIITA mutants to increase Gag-Pol levels relative to Gag and, interestingly, Gag-Pol made up approximately 13, 20 and 15% of all Gag polyproteins in GTP2, GTP3 and L1035P –expressing cells, respectively. This result further demonstrates a novel transcription-independent role for cytoplasmic CIITA during HIV infection, where CIITA enhances Gag-Pol protease expression, which subsequently enhances virus maturation and infectivity.

## Discussion

Finzi *et al*. (2006) demonstrated that transient overexpression of HLA-DR heterodimers in HEK-293 cells lead to redistribution of Gag and intracellular accumulation of infectious virus in multivesicular bodies (MVBs) [16]. We speculated that complete expression of the antigen presentation pathway, through expression of CIITA, would alleviate this phenotype in virus producer cells. Interestingly, we noticed two phenomena: i) CIITA did not induce intracellular retention of Gag or impair virus release in producer cells despite expression of HLA-DR and ii) the virus released from cells expressing CIITA was significantly more infectious. Upon investigation of CIITA-mediated alleviation of HLA-DR-induced Gag retention, we found that this effect was not due to the lack of Ii or HLA-DM (key components of antigen presentation), as had been expected, nor was it a consequence of Rab4B expression in these cells. Further, when lysine residues in the HLA-DR chain were mutated or both of the HLA-DR chain cytoplasmic tails were truncated, Gag was still retained and virus release inhibited. In addition, when CIITA was expressed in conjunction with HLA-DR this effect could not be alleviated, suggesting that Gag retention is a consequence of HLA-DR overexpression. Indeed, retention of Gag is directly correlated to levels of HLA-DR expression. Further, we did not observe Gag retention when class II antigen presentation pathway genes were expressed at endogenous levels via CIITA expression. Overexpression of HLA-DM, which is structurally similar to HLA-DR also induced Gag retention, suggesting that retention of Gag is a likely consequence of altered trafficking of overexpressed class II antigen presentation pathway components rather than a physiologically relevant phenomenon.

Despite observing similar HLA-DR and Gag retention/reduced virus release effects as Finzi *et al*.(2006), our results differed in that we did not observe the requirement for intact HLA-DR α and β-chain cytoplasmic tails for the induction of Gag retention. In our hands, when the ubiquitinatible lysine residues in the HLA-DR β-chain were mutated or the cytoplasmic tails of the HLA-DR dimer were completely removed, there was no alleviation of Gag retention or virus release. Beyond the obvious differences between the previous work [8] and our own (i.e. provirus construct and cell type), our mutant data may differ from theirs because arginine residues were substituted for lysine 215 and tyrosine 220 residues in the HLA-DR α and β chain, respectively, in order to ensure stabilization of the truncated HLA-DR molecule at cellular membranes. These differences in mutant constructs may potentially explain discrepancies in our results.

We also observed that expression of HLA-DM was sufficient to induce Gag retention and impede virus release from cells. However, Finzi *et al*.(2006) did not observe retention in the presence of HLA-DM or HLA-DO [8]. This difference may be explained by their use of a bicistronic construct for expression of the HLA-DM heterodimer; however, this strategy was also used to express HLA-DR, which still induced retention [8]. Irrespective of these differences, Gag retention and loss of virus release correlates with increasing HLA-DR expression. These results do not exclude the possibility that HLA-DR and Gag trafficking may be linked. Indeed, we have demonstrated that Gag co-immunoprecipitates with HLA-DR from CIITA expressing cells and this interaction is independent of the cytoplasmic tails of HLA-DR (data not shown). Further, other HIV proteins may be linked to class II trafficking. Stumptner-Cuvelette *et al*.(2003) and Chaudhry *et al*.(2009), have independently demonstrated that HIV Nef induces internalization of surface class II in epithelial and monocytic cell lines, respectively [37], [64]. However, we did not observe downregulation of HLA-DR from the cell surface, following transfection of the HIV genome into CIITA-expressing epithelial cells (data not shown) or HIV infection of CIITA-expressing CD4+ T cells (unpublished data). Further, Gluschankof and Suzan demonstrated that expression of Gag-Pol restored HLA-DR presence at the cell surface in the H78-C10.0 line, a T cell clone deficient for surface HLA-DR expression [40]. Collectively, our work and that of others suggests a link between HLA-DR and Gag trafficking, where localization may be cell-type dependent.

One of the more interesting findings of this study is that while overall viral titers from CIITA-expressing cells decrease the infectivity of these particles is significantly enhanced. Increased infectivity was due to improved virion maturation in a viral protease-dependent manner. Not only was processing to capsid p24 more complete in CIITA expressing cells, but vector-only control cells and those only expressing HLA-DR contained increased levels of processing intermediates. The presence of higher levels of processing intermediates in virus produced in these cells may help explain the reduced infectivity, as studies in both MLV [65] and HIV [7] demonstrate that partially cleaved Gag products act transdominantly to reduce virion infectivity through reduced reverse transcription of viral RNA, despite the presence of functional Reverse Transcription polymerase [7].

Next we demonstrated that CIITA increased Gag processing through the viral protease and evaluated whether this enhancement was a consequence of CIITA-mediated transcriptional activation. The CIITA L1035P mutant, which fails to translocate to the nucleus [58], demonstrated increased Gag processing and virus release compared to vector-only control, suggesting a cytoplasmic role for CIITA in the later stages of the HIV infection cycle. This does not preclude the possibility that an undetectable level of CIITA L1035P might translocate to the nucleus. However, no L1035P was detected in the nucleus after a 24 hour treatment with the nuclear export inhibitor, leptomycin B [58]. Further, the predominantly cytoplasmic GTP-binding CIITA mutants, GTP2 and GTP3, had a similar increase in infectious virus release versus vector-only expressing cells.

Previously, it was demonstrated that increased Gag-Pol levels severely impair virion infectivity through disruption of RNA genome dimerization [62]. Further, HIV protease is known to be toxic to cells as it leads to the production of the novel Procaspase 8 cleavage product, Casp8p41, which induces apoptosis through loss of mitochondrial membrane potential [66]. Therefore, we would expect that virions from CIITA-expressing cells would be reduced in titer and infectivity, as there is increased Gag-Pol and protease. However, while overall viral particle numbers (as measured by p24 ELISA) from CIITA expressing cells were decreased, the infectivity of these virions was significantly increased when calculated by infectious units per pg p24. Interestingly, at 0.6 nM Lopinavir, the infectivity of virions from CIITA-expressing cells increased over vehicle-treated controls, despite reduced Gag processing. Further, the mutants of CIITA, which produced the highest level of Gag-Pol, did not demonstrate a linear increase in Gag processing or virion infectivity, which may be explained by previous work demonstrateing that increased Gag-Pol levels impairs viral infectivity [61], [62]. Therefore it is likely that CIITA is capable of increasing Gag-Pol levels to a point which can impede virus maturation, albeit not enough to reduce it to levels observed from vector-only expressing cells. Therefore, overall infectivity of virions is increased from cells expressing CIITA despite an altered Gag to Gag-Pol ratio. Future studies should focus on how CIITA can increase this ratio without severely impairing virus release as well the novel mechanism by which CIITA increases Gag-Pol levels. Preliminary studies in this laboratory suggest that CIITA enhancement Gag-Pol may be due to increased ribosomal frameshifting (data not shown).

Finally, it is tempting to speculate that CIITA, which is expressed upon CD4+ T cell activation and increases viral protease levels, may also contribute to Casp8p41-mediated apoptosis, which may link CIITA to the decimation of T cells in the GALT during primary viremia [67]. Thymic epithelial cells [68], cervical epithelial cells [69], human colonic epithelial cells [70] and oral keratinocytes (normal human oral epithelial cells) [71] have all demonstrated *in vitro* the capability of being productively infected with HIV. Infection of epithelial cells provides potential *in vivo* significance to this study, especially considering that thymic epithelial cells constitutively express MHC class II (and thus CIITA) [28]. In addition, most other cells can be stimulated to express CIITA in the presence of IFN-γ (i.e. a pro-inflammatory environment), which is induced during HIV infection of the GALT [72]. Overall, this study demonstrates that the function of CIITA may be more broad than previously thought. Given this previously undescribed role in enhancement of virion maturation, the precise consequences of CIITA expression during the HIV replicative cycle may provide rationale for the development of novel antiviral therapeutics.

## Materials and Methods

### Cell Culture

A293T cells [73] were maintained as previously described [74] and CIITA-stable A293T cell lines were generated by co-transfecting PVU I linearized pDNA3.FLAG.CIITA8 [75] and pCMV4His, a mammalian selection vector which encodes the histidinol dehydrogenase gene under control of the CMV promoter into the cells. Stable clones were selected in DMEM medium containing 5 mM L-histidinol (Sigma-Aldrich., St. Louis, MO) and cloned by limiting dilution assay. GHOST cell medium was supplemented with 0.2 mg/ml G418, 0.1 mg/ml hygromycin B and 1 µg/ml puromycin (Sigma) as previously described [76].

### Cloning and transfections

Using cDNA reverse transcribed from A293T-CIITA RNA, we amplified HLA-DRα, HLA-DRβ1 and HLA-DRβ5 sequences (GenBank accession nos. NM019111, NM002121, and NM002125, respectively), using primers containing forward restriction site XbaI and the reverse restriction site HindIII (Table S1). The haplotyping of HLA-DR isotypes expressed by A293T cells was determined by the Transplantation Immunology Lab, Albany Medical College. Primers were designed to include an intact Kozak sequence upstream of the translation start site. HLA-DRα and β5 plasmids were then used for site-directed mutagenesis using primers indicated (Table S1). Transient transfections of all plasmids, including: pDNA3.FLAG.CIITA8 [75], CIITA-GTP2 and -GTP3 [56] and CIITA -L1035P [58], p33-143 (coding for the p33 and p35 isoforms of invariant chain–a kind gift provided by Dr. Eric O. Long, NIAID), HLA-DMα and β (pMCFR-PAC and pDMβ/MCFR- kindly provided by Dr. Lisa K. Denzin, Sloan-Kettering Cancer Center), eGFP-Rab4B (kindly provided by Dr. Marci Scidmore, Cornell University), and HIV Gag-iGFP [77] (kindly provided by Dr. Benjamin Chen, Mount Siani School of Medicine) were performed using a 3∶1 ratio Fugene HD Transfection Reagent to DNA in serum-free media as suggested by the manufacturer (Roche, Indianapolis, IN). Virus plasmid DNA provided half of total DNA used in transfection reactions.

NIH 3T3 Balb/c cells are transfected with 1.5 µg CIITA and 4.5 µL FuGene HD (7∶2 ratio of FuGene to DNA for optimal cells growth) and selected for with L-histidionol for two weeks and analyzed for MHC II expressing using fluorescence-activated cell sorter (FACS) with PE-conjugated mouse IgG2a (Cedar Lane) against I-A^d^ and clones were selected by limiting dilution. These cells were co-transfected with p32cDNA and pcHIV PAL (which contains all HIV genes except the LTR sequences) at a 2∶1 ratio. The p32cDNA was donated by the Peterlin laboratory [59]. Both MHC II expressing VLPs and MHC II-negative VLPs were produced and concentrated 10-fold in a 100 K molecular weight cut-off filter tube (Millipore) for 15 minutes at 4000 rpm prior to titering via HIV-1 p24 CA Antigen Capture Assay Kit were performed following the manufacturer's instructions (NIH AIDS Research and Reference Reagent Program).

### RNA Extraction

Cytoplasmic RNA from 48 hr cultures of each cell type was collected using the RNeasy Mini Kit according to manufacturer's instructions (Qiagen, Inc., Valencia, CA).

### RT-PCR

1ug of DNA-free RNA was run out on a non-denaturing gel to ensure equal concentrations of each sample followed by reverse transcription of 1 µg of each RNA sample using the iScript cDNA synthesis kit, following manufacturer's instructions (BioRad, Hercules, CA). 50 ng of cDNA was used to amplify *HLA-DM, CIITA, gapdh*, and *Ii* from each cell type to confirm expression. Forward and reverse primers sequences used in RT-PCR experiments indicated in Table S1.

### Flow Cytometry

Cells were analyzed for HLA-DR expression as previously described [56]. Briefly, cells were incubated with murine clone L243 [78] hybridoma (ATCC, Manassas,VA) supernatant diluted 1∶10 in PBS/2% FBS for 30 minutes on ice, washed, and then incubated with goat- anti-mouse Alexa-Fluor 488- or Alexa-Fluor 647-conjugated secondary antibody (Molecular Probes/Invitrogen) diluted 1∶100 in PBS/2% FBS for 20 minutes on ice in the dark. Alternatively, the cells were incubated with L243 monoclonal antibody conjugated to AlexaFluor 488 or 647 (BioLegend) diluted 1∶50 in PBS/2% FBS for 40 minutes on ice in the dark. Cells were then analyzed with the FACSCanto (BD Biosciences, San Jose, CA) and further analyzed with Flo-Jo 7.2.2 software (Tree Star, Inc, Ashland, OR). For Gag retention experiments, live cells were gated, followed by gating of DR+ cells and then the percentage of Gag and Gag^hi^ cells were gated. Microscopy on the same samples was performed using Leica L2 microscope (Leica Microsystems Wetler GmbH, Wetzler, Germany).

### Western blotting

(1∶000) of mouse anti-human Rab4 (BD, Franklin Lakes, NJ), rabbit anti-human β-actin (Cell Signaling Tech., Danvers, MA), (1∶200) HIV-1 p24 hybridoma supernatant (183-H12-5C)[79], (1∶10,000, NIH AIDS Research and Reference Reagent Program, Germantown, MD) and (1∶1000) mouse anti-FLAG M2 (Sigma). Secondary antibodies used: HRP conjugated, Goat anti-Mouse IgG and Goat anti-Rabbit IgG (Zymed grade) or (Invitrogen, Carlsbad, CA). Densitometric analysis was performed as previously described [80] and the percentage of total HIV-1 α CA p24 (183-H12-5C) [79] reactive bands was used as a measure of Gag processing [81].

### Intracellular Gag staining

10^6^ cells were permeabilized with IntraPrep Permeabilization Reagent (Beckman-Coulter, Fullerton, CA), following manufacturer's instructions. Gag was stained with FITC-conjugated FH190-1-1 (Beckman-Coulter) for approximately 15 m prior to analysis and data interpretation as performed above, with the exception of the percentage of Gag+ cells was determined after gating on HLA-DR+ cells.

### Virus purification and titering

Virions were purified using the µMACS Streptavidin Kit (Miltenyi Biotec Inc., Auburn, CA.), according to the manufacturer's instructions using biotinylated-L243 (Biolegend). GHOST assay [82] to determine infectious virus production and p24 ELISAs to determine virus particle release using the HIV-1 p24 CA Antigen Capture Assay Kit were performed following the manufacturer's instructions (NIH AIDS Research and Reference Reagent Program).

### Protease inhibition

Cells were transfected with either pcCIITA or empty pcDNA vector 4.5 hours prior to Lopinavir (NIH AIDS Research and Reference Reagent Program) or DMSO (vehicle-only) treatment at indicated concentrations. Virus and cell supernatants were collected approximately 17 hours post-treatment. For determination of p160Gag-Pol to p55Gag ratios, producer cells were transfected with CIITA constructs (pcCITIA, GTP2, GTP3, L1035P) or vector only control (pcDNA) and then transfected with pNL4-3 16 hours later, prior to being treated with 80 nM Lopinavir. Cell lysates were used to monitor the levels of Pr55Gag to Pr160Pol via western blotting with HIV-1 α CA p24 (183-H12-5C) after 17 hours.

## Supporting Information

Table S1 Primers used in this study.
(0.06 MB DOC)

Figure S1 Gene expression analysis via semi-quantitative PCR was performed on A293T cells transfected with the indicated constructs (A), where the Hut78 T cell line served as a positive control. Expression of HLA-DR on the surface of cells transfected with the indicated plasmids was assessed by flow cytometry (B).
(0.71 MB DOC)

Figure S2 Virus titers were determined at 48 h.p.t. following transfection with indicated plasmids and HIV_NL4-3_, as determined by GHOST infectivity assays and p24 ELISA. Standard deviation of the mean for 3 independent experiments is presented.
(0.07 MB DOC)

Figure S3 Representative blot of Gag processing in virions from cells treated with indicated concentrations of Lopinavir, where Vehicle  =  vehicle only (DMSO).
(0.41 MB DOC)

Figure S4 Gag processing of virions from cells transfected with indicated constructs and HIV_NL4-3_ was measured via western blotting with antibody against CAp24 followed by densitometric analysis of Gag cleavage products.
(0.07 MB DOC)

Figure S5 p32cDNA and pcHIV PAL were cotransfected into the indicated NIH 3T3 Balb/c cells. At 3 d.p.t. virus containing supernatants were used for titering via p24 ELISA assay. Data is representative of three independent experiments, where error bars indicate standard error.
(0.03 MB DOC)
