# Supplementary material for: Class II Transactivator (CIITA) Enhances Cytoplasmic Processing of HIV-1 Pr55Gag
Source: PLoS One. 2010 Jun 24;5(6):e11304. doi: 10.1371/journal.pone.0011304 (PMC2892040; doi:10.1371/journal.pone.0011304)
Supplement: Table S1 — Primers used in this study. (0.06 MB DOC) [file pone.0011304.s001.doc]

| **Gene** | | **Primer** | | **Sequence**  **5’ – 3’** | | **Amplicon size** | **Source** | |
| --- | --- | --- | --- | --- | --- | --- | --- | --- |
| **Cloning** | | | | | | | | |
| HLA-DR α | | | Forward | | ttcttttattctagactgttctgc | 875bp |  | |
|  | | | Reverse | | ttctctctaagcttgaaacaccat |  |  | |
| HLA-DRβ0101 | | | Forward | | ttgcctgcttctctagaccctggtcctgtc | 920bp |  | |
|  | | | Reverse | | taactgccaagcaggaaagcttttcattct |  |  | |
| HLA-DRβ0501 | | | Forward | | catcatgaatctagaccagcatggt | 901bp |  | |
|  | | | Reverse | | aataagagccaagcaggaaagcttt |  |  | |
| DRβK225R | | |  | | ATCTACTTCAAGAATCAG[C/G][C/G]AGGGCACTCTGGACTTCAC |  |  | |
|  | | |  | | 5’GTGAAGTCCAGAGTGCCCT[C/G][C/G]CTGATTCTTGAAGTAGAT |  |  | |
| DRβK222/225R* | | |  | |  |  |  | |
|  | | |  | |  |  |  | |
| DRαΔcyto (Lys215ArgSTOP) | | | Forward | | ACCATCTTCATCATCCGGTGATTGCGCAAAAGCAATGCAGCAGAACGC |  |  | |
|  | | | Reverse | | GCGTTCTGCTGCATTGCTTTTGCGCAATCACCGGATGATGAAGATGGT |  |  | |
| DRβΔcyto (Tyr220ArgSTOP) | | | Forward | | GGGGCCGGGCTATTCATCCGCTAAAGGAATCAGCGAGGACAC |  |  | |
|  | | | Reverse | | GTGTCCTCGCTGATTCCTTTAGCGGATGAATAGCCCGGCCCC |  |  | |
| **RT-PCR** | | | | | | | | |
| Invariant Chain | | Forward | | GAATGCTGACCCCCTGAAGGTGTA | | 586 bp** | [1] | |
| (CD74) | | Reverse | | GGGGGCTGAAGGGAGCAAGAAAGC | | 396 bp*** |
| HLA-DMα | | Forward | | ACT TTT CCC AGA ACA CTC GG | | 341 bp | [2] | |
|  | | Reverse | | CTG GAA GCT GAG TCC ATC G | |  |
| CIITA | | Forward | | AACCCGACACAGACACCATCAACT | | 107 bp |  | |
|  | | Reverse | | GTCCAGTTCCGCGATATTGGCATA | |  |  | |
| GapDH | | Forward | | CAAAAGGGTCATCATCTCTGC | | ~200 bp | [3] | |
|  | | Reverse | | CAGGGGCCATCCACAGTCTTC | |  |
|  | *Sequencing results confirmed a clone with correct K225R mutation as well as another clone with a spontaneous K222R mutation, thereby providing substitutions in both ubiquitinatible lysine residues of the HLA-DR β chain (K222/225R). | | | | | | |  |
|  | ** isoform p41, ***isoform p33 | | | | | | |  |

1. Barrera CA, Beswick EJ, Sierra JC, Bland D, Espejo R, et al. (2005) Polarized expression of CD74 by gastric epithelial cells. J Histochem Cytochem 53: 1481-1489.

2. Hershberg RM, Framson PE, Cho DH, Lee LY, Kovats S, et al. (1997) Intestinal Epithelial Cells Use Two Distinct Pathways for HLA Class II Antigen Processing. J Clin Invest 100: 204-215.

3. Morrison TE, Mauser A, Wong A, Ting JPY, Kenney SC (2001) Inhibition of IFN-[gamma] Signaling by an Epstein-Barr Virus Immediate-Early Protein. Immunity 15: 787-799.
