# Supplementary figures and images for: Class II Transactivator (CIITA) Enhances Cytoplasmic Processing of HIV-1 Pr55Gag
Source: PLoS One. 2010 Jun 24;5(6):e11304. doi: 10.1371/journal.pone.0011304 (PMC2892040; doi:10.1371/journal.pone.0011304)

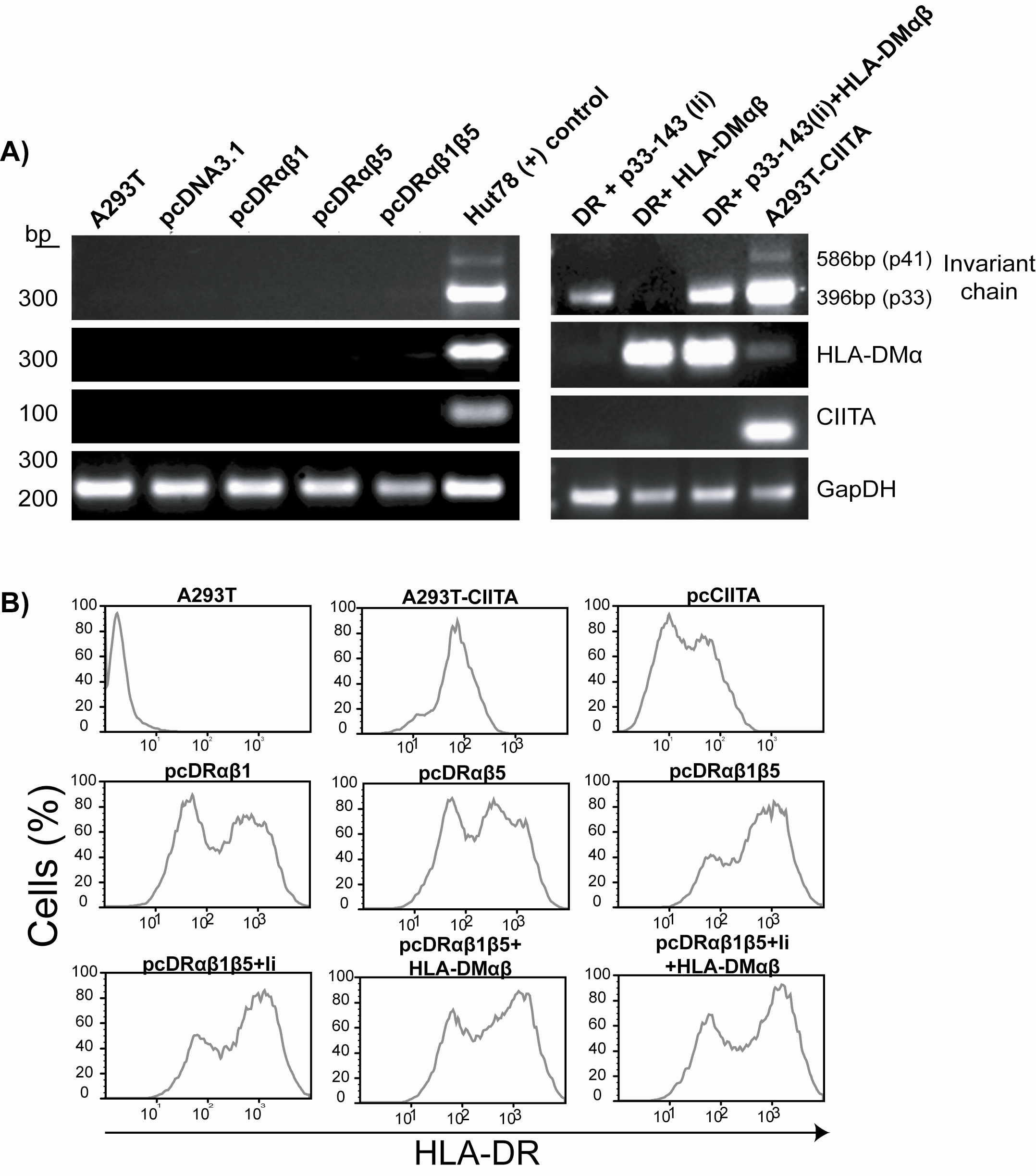

Supplement: Figure S1 — Gene expression analysis via semi-quantitative PCR was performed on A293T cells transfected with the indicated constructs (A), where the Hut78 T cell line served as a positive control. Expression of HLA-DR on the surface of cells transfected with the indicated plasmids was assessed by flow cytometry (B). (0.71 MB DOC) [file pone.0011304.s002.doc]

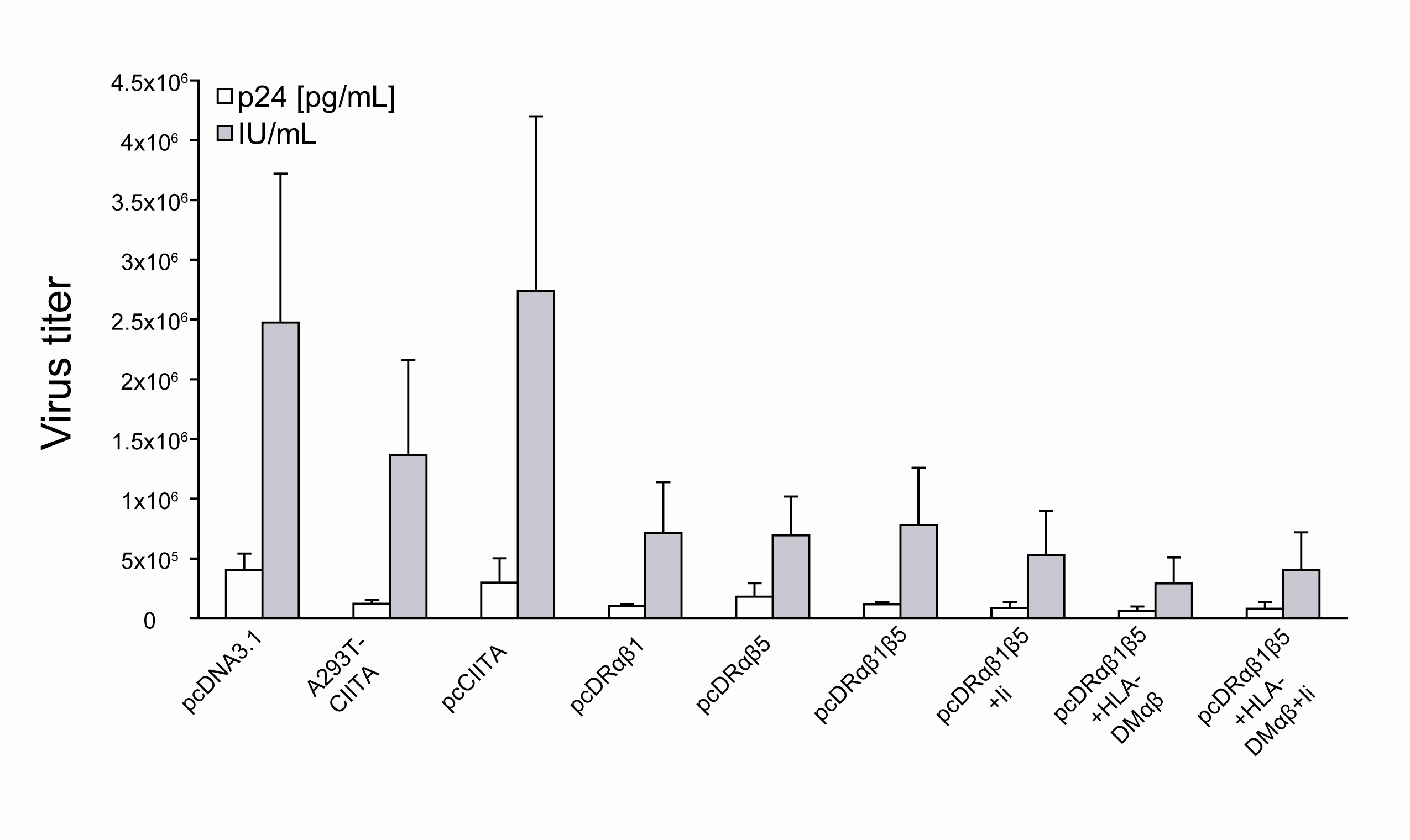

Supplement: Figure S2 — Virus titers were determined at 48 h.p.t. following transfection with indicated plasmids and HIVNL4-3, as determined by GHOST infectivity assays and p24 ELISA. Standard deviation of the mean for 3 independent experiments is presented. (0.07 MB DOC) [file pone.0011304.s003.doc]

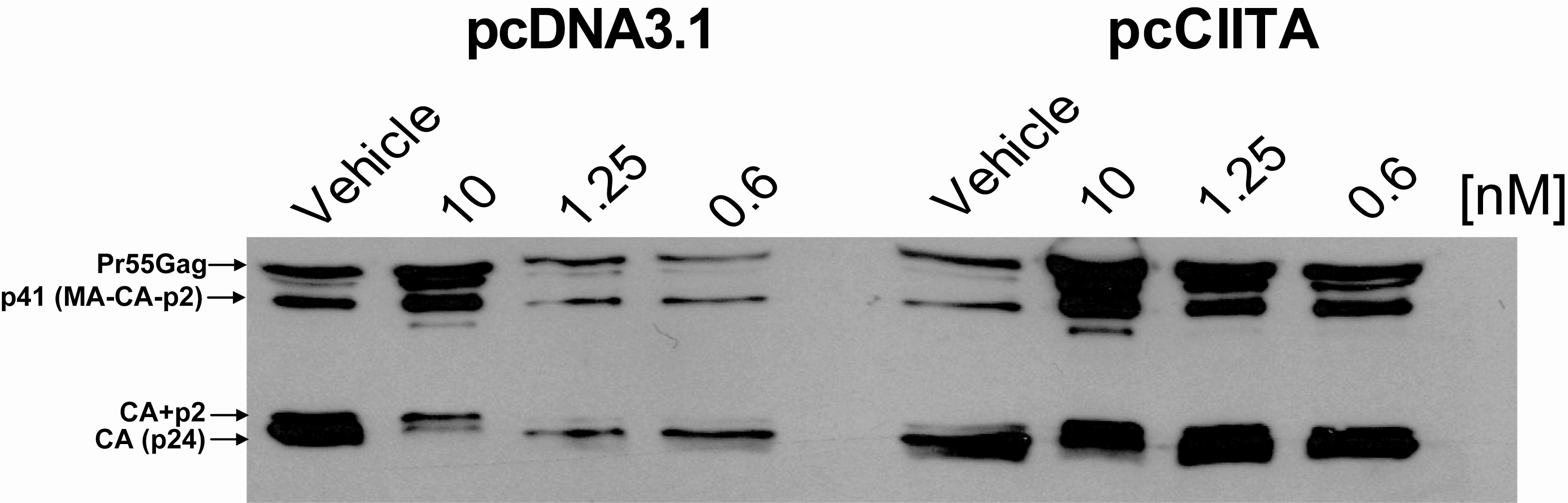

Supplement: Figure S3 — Representative blot of Gag processing in virions from cells treated with indicated concentrations of Lopinavir, where Vehicle = vehicle only (DMSO). (0.41 MB DOC) [file pone.0011304.s004.doc]

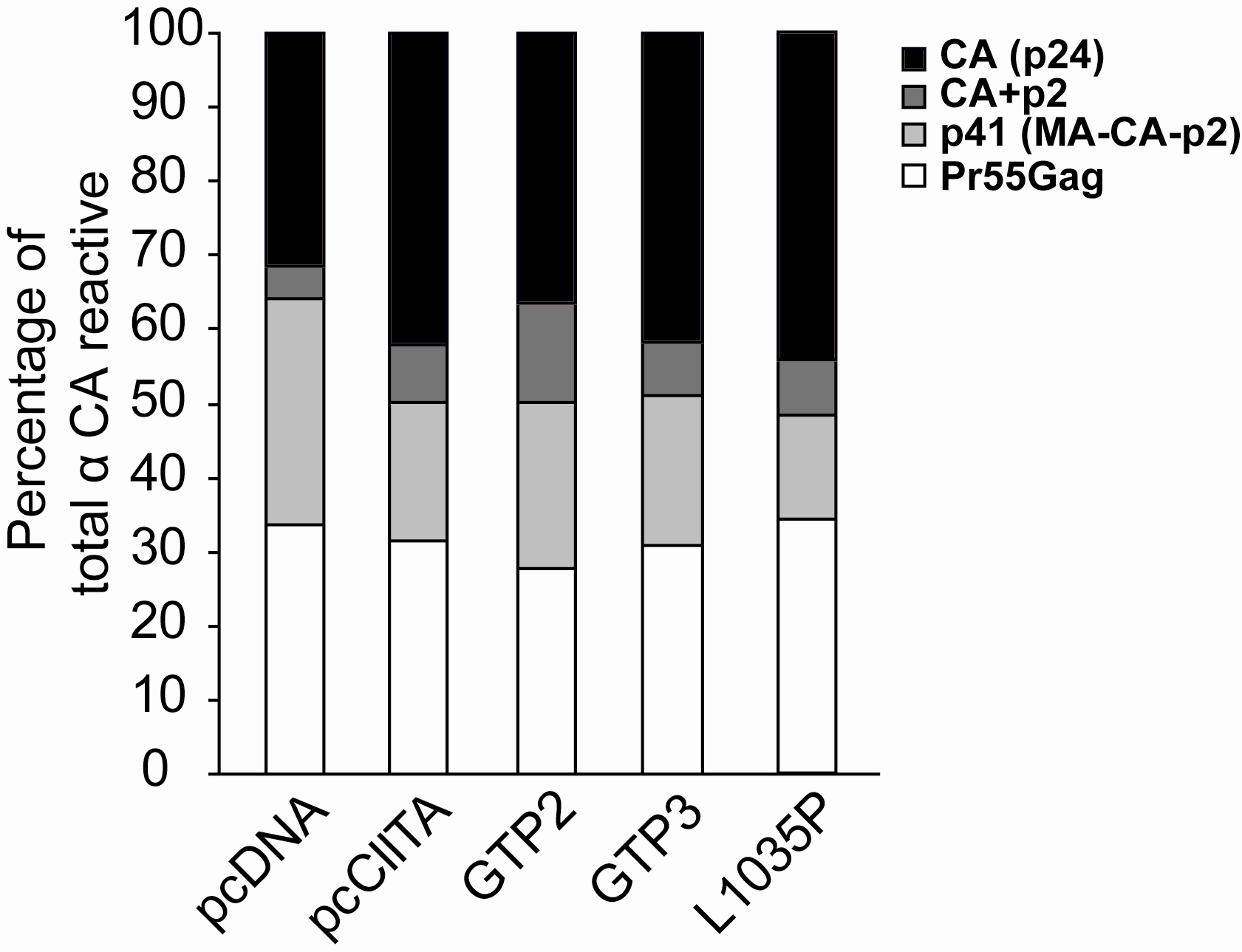

Supplement: Figure S4 — Gag processing of virions from cells transfected with indicated constructs and HIVNL4-3 was measured via western blotting with antibody against CAp24 followed by densitometric analysis of Gag cleavage products. (0.07 MB DOC) [file pone.0011304.s005.doc]

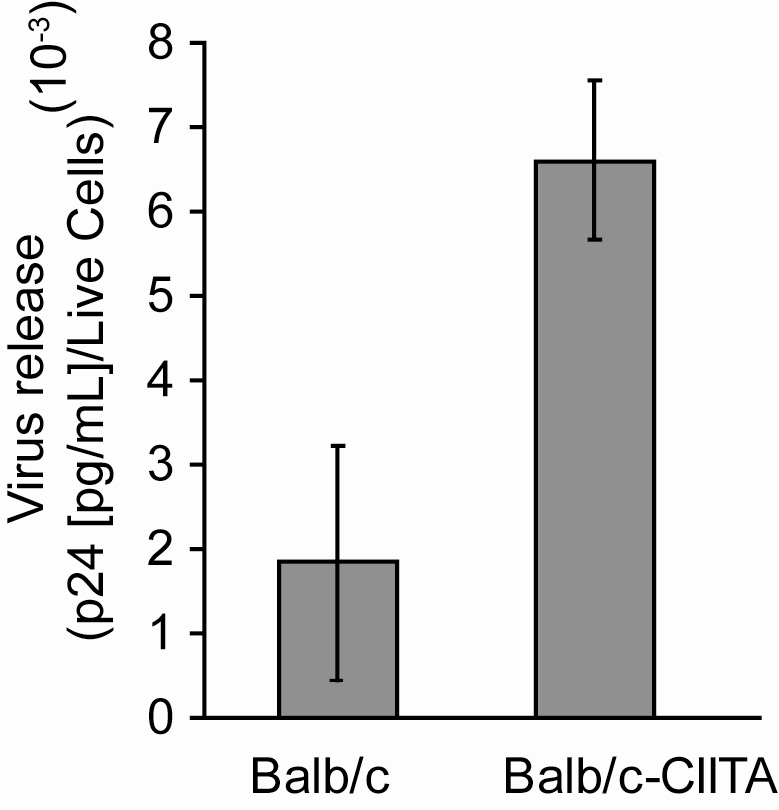

Supplement: Figure S5 — p32cDNA and pcHIV PAL were cotransfected into the indicated NIH 3T3 Balb/c cells. At 3 d.p.t. virus containing supernatants were used for titering via p24 ELISA assay. Data is representative of three independent experiments, where error bars indicate standard error. (0.03 MB DOC) [file pone.0011304.s006.doc]
